# Survey of dysphagia and related medications in nursing home residents using the Eating Assessment Tool (EAT-10) applied by community pharmacists: A single-center retrospective study

**DOI:** 10.1186/s40780-025-00447-0

**Published:** 2025-05-07

**Authors:** Norio Watanabe, Minami Ebisujima, Takahiro Ichihara, Motozumi Ando, Masami Kawahara

**Affiliations:** 1 Clinical Pharmacy and Sciences, School of Pharmacy, Aichi Gakuin University, 1-100, Kusumoto-Cho, Chikusa-Ku, Nagoya, Aichi 464-8650 Japan; 2 COSMOS CHOUZAI Pharmacy Co., Ltd, 5-4-14, Meieki, Nakamura-Ku, Nagoya, Aichi 450-0002 Japan

**Keywords:** Community pharmacists, EAT-10, Nursing homes, Antipsychotics, Drug-induced dysphagia

## Abstract

**Background:**

This study aimed to assess the prevalence of dysphagia among nursing home residents and to examine the association between medication use and impaired swallowing function.

**Methods:**

Between January and December 2023, we conducted a retrospective survey across 14 nursing homes visited by community pharmacists. Swallowing function was evaluated using the Eating Assessment Tool-10 (EAT-10) questionnaire. Residents were categorized into two groups: those with reduced swallowing function (EAT-10 score ≥ 3) and those with normal swallowing function (EAT-10 score < 3). The association between medication use and swallowing function was analyzed using univariate and multivariate logistic regression models.

**Results:**

Significant differences were observed between the reduced (*n* = 101, 36.9%) and normal swallowing function (*n* = 173, 63.1%) groups in terms of age (*P* = 0.022), body mass index (BMI) (*P* < 0.001), and nursing care level (*P* < 0.001). Propensity score matching was performed to adjust for confounding factors, yielding 72 matched pairs. Analysis of the matched cohort revealed a significant association between antipsychotic use and reduced swallowing function (odds ratio [OR], 2.600; 95% confidence interval [CI], 1.210–5.560; *P* = 0.014).

**Conclusions:**

This study identified a significant association between antipsychotic drug use and reduced swallowing function. Medication reviews incorporating assessments of swallowing function may help mitigate the risk of aspiration. Further prospective studies are warranted to validate these findings and to clarify causal relationship between medication use and swallowing dysfunction.

## Background

As Japan’s population continues to age, the incidence of choking caused by aspiration and aspiration pneumonia is increasing among older adults. Consequently, the importance of providing feeding and swallowing support in medical institutions and nursing homes has been emphasized [1, 2]. Drug-induced dysphagia has been identified as a potential cause of dysphagia, with antipsychotic use, in particular, being associated with reduced swallowing function. However, a comprehensive understanding of this issue remains limited [3]. In addition to age-related decline in swallowing function, factors such as polypharmacy-related adverse events and reduced drug metabolism and excretion capacity, particularly when six or more categories of drug combined, have been reported to increase the risk of adverse events [4–6]. Specifically, extrapyramidal symptoms induced by antipsychotics can lead to side effects, such as reduced pharyngeal muscle function, whereas the sedative and muscle-relaxing properties of anxiolytics and hypnotics may suppress the swallowing reflex. Additionally, anticholinergic medications can cause oral dryness, further exacerbating dysphagia [7, 8].

In Japan and internationally, prescription suitability screening tools designed to amend inappropriate prescriptions are considered valuable for reducing the risk of adverse drug-related outcomes. However, these tools have been underutilized in studies on drug-induced dysphagia. Many patients with neurodegenerative diseases such as stroke, dementia, and Parkinson’s disease struggle to improve their swallowing function through exercise and feeding support alone. As certain medications, including antipsychotics, may further impair swallowing function, their use must be carefully adjusted [9].

Swallowing function is commonly assessed using methods such as the modified water Swallow Test and Food Tests [10]. However, these techniques require specialized training and may pose a risk of aspiration. Questionnaire-based assessment tools have attracted considerable attention as safe and more convenient alternatives. The Eating Assessment Tool (EAT-10), a standardized questionnaire consisting of 10 items, is widely used in hospitals and nursing homes to evaluate swallowing function [11, 12].

To better understand drug-induced dysphagia, it is essential to investigate swallowing function and its association with medication use. However, few studies have used the EAT-10 questionnaire for this purpose. Conducting research using the EAT-10 could help identify medications that contribute to the risk of dysphagia and inform suitable interventions.

For patients with reduced swallowing function who have difficulty swallowing tablets or capsules, pharmacists can recommend interventions such as the simple suspension method, use of thickeners, or modifications to dosage forms. However, information on the swallowing function is primarily provided by physicians, nurses, or nursing home staff, and pharmacists have limited opportunities to participate in the assessment processes. By actively evaluating swallowing function and using the findings to guide medication use, pharmacists can help reduce the risk of aspiration and support safer medication administration.

Surveys conducted in hospitals and nursing homes have also revealed an implementation rate of less than 30% for swallowing function assessments and screening of medications with potential links to reduced swallowing function, highlighting the challenges caused by a lack of interprofessional cooperation [13]. Pharmacists involved in home care settings typically evaluate the swallowing function, share their findings, and collaborate with other healthcare professionals. Strengthening this cooperation is essential to reduce the risk of choking due to aspiration and aspiration pneumonia.

This retrospective study aimed to use the EAT-10 questionnaire to assess the prevalence of dysphagia among residents of nursing homes visited by community pharmacists, and to investigate the relationship between swallowing function and medication use.

## Methods

### Survey period and population

Between January and December 2023, we retrospectively surveyed residents of 14 nursing homes visited by pharmacists employed by COSMOS CHOUZAI Pharmacy Co., Ltd. Patients with severe cognitive impairment or those using gastrostomy feeding were excluded because of anticipated difficulties in administering the EAT-10 questionnaire. Severe cognitive impairment was defined as (1) a physician's diagnosis of profound dementia or (2) a consistent inability to understand or communicate during daily activities, as documented in nursing care records.

As this was a retrospective observational study, an a priori sample size calculation was not performed. Instead, we included all residents who met the eligibility criteria from the participating nursing homes during the study period to minimize selection bias.

### Survey method

In addition to reviewing pharmacy dispensing records and medication histories, we collected data on patient age, sex, body mass index (BMI), nursing care level, and the presence of medical conditions known to cause dysphagia (stroke, Parkinson’s disease, or dementia). We also recorded the medications potentially associated with reduced swallowing function, including antipsychotics, anxiolytics/hypnotics, anticholinergics, antiemetics/antiulcer agents, and central muscle relaxants. Finally, EAT-10 scores were reported. The EAT-10 assessments were conducted when patients were deemed alert and cooperative by pharmacists and nursing staff. To minimize inter-rater variability, EAT-10 assessments were performed by trained community pharmacists who had received standardized training in administering the questionnaire.

The medications were selected based on the 2024 Clinical Guidelines for Dysphagia from the Japanese Society of Otorhinolaryngology–Head and Neck Surgery [7] and a report by Kurata [8].

Patients with an EAT-10 score ≥ 3 were classified into the reduced swallowing function group, and those with a score < 3 were classified into the normal swallowing function group.

### Statistical analysis

Univariate analysis was conducted to investigate the presence or absence of dysphagia based on EAT-10 scores and related background factors. Nominal variables were compared using Fisher’s exact test, whereas continuous variables were analyzed using the Mann–Whitney U test. Statistical significance was set at *p* < 0.05 in all analyses. Propensity score matching was performed to adjust for potential confounding factors. Propensity scores were calculated using logistic regression analysis, with age, sex, BMI, nursing care level, and underlying conditions (stroke, Parkinson's disease, dementia) as explanatory variables and swallowing function status (reduced vs. normal) as the outcome variable. Patients were matched in a 1:1 ratio using the nearest neighbor method with a caliper width of 0.2 to ensure accurate matching.

To analyze the relationship between medication use and reduced swallowing function, variables with a *p*-value < 0.15 in the univariate analysis were included in a multivariate logistic regression analysis.

All statistical analyses were performed using EZR version 1.54 (Saitama Medical Center, Jichi Medical University, Saitama, Japan), which is a graphical user interface for R (R Foundation for Statistical Computing, Vienna, Austria) [14]. EZR is a modified version of the R commander, designed to add statistical functions frequently used in biostatistics.

## Results

### Patient characteristics (full population)

Among 287 residents initially considered for inclusion, 13 were excluded due to severe cognitive impairment and 3 were excluded because they were receiving gastrostomy feeding. Consequently, 274 residents were included in the final analysis. This study included 274 nursing home residents with a median age of 85.0 years (interquartile range [IQR]: 76.0–90.0). Of these, 87 (31.8%) were male and 187 (68.2%) were female. Regarding nursing care level, seven residents (2.6%) “required assistance,” whereas 267 (97.4%) “required nursing care.” Among the conditions associated with dysphagia, dementia was the most common, affecting 103 residents (37.6%), followed by stroke, affecting 50 patients (18.2%).

Regarding medication use, 84 residents (30.7%) were taking antipsychotics and 69 residents (25.2%) used anxiolytics/hypnotics, both of which are associated with reduced swallowing function. Regarding the method of medication administration, none of the patients used the simple suspension method.

Based on the EAT-10 questionnaire score, 101 residents (36.9%) were classified into the reduced swallowing function group and 173 residents (63.1%) into the normal swallowing function group. These groups showed significant differences in terms of age, BMI, and nursing care level (Table 1). Residents in the reduced swallowing function group were older (median 86.0 years IQR: 79.0–90.0 vs. 83.0 years IQR: 75.0–90.0; *P* = 0.022), had a lower BMI (median 18.9 kg/m^2^ IQR: 17.6–20.7 vs. 20.3 kg/m^2^ IQR: 18.6–22.5; *P* < 0.001), and had higher nursing care levels (P < 0.001) than those in the normal swallowing function group.

**Table 1:** Patient characteristics (full population)

		Full population (n = 274)		EAT-10 Questionnaire				
				Reduced swallowing function group (*n* = 101)		Normal swallowing function group (*n* = 173)		*p*-value
Age		85.0	[76.0–90.0]	86.0	[79.0–90.0]	83.0	[75.0—90.0]	0.022^a^
Sex	Male	87	(31.8)	32	(31.7)	55	(31.8)	1.000^b^
	Female	187	(68.2)	69	(68.3)	118	(68.2)	
BMI (kg/m^2^)		19.7	[18.1–21.9]	18.9	[17.6–20.7]	20.3	[18.6—22.5]	< 0.001^a^
Nursing care level	Requiring assistance 1	1	(0.4)	0	(0)	1	(0.6)	< 0.001^b^
	Requiring assistance 2	6	(2.2)	1	(1.0)	5	(2.9)	
	Requiring nursing care 1	17	(6.2)	3	(3.0)	14	(8.1)	
	Requiring nursing care 2	39	(14.2)	5	(5.0)	34	(19.7)	
	Requiring nursing care 3	74	(27.0)	21	(20.8)	53	(30.6)	
	Requiring nursing care 4	102	(37.2)	45	(44.6)	57	(32.9)	
	Requiring nursing care 5	35	(12.8)	26	(25.7)	9	(5.2)	
Disease causing dysphagia	Stroke	50	(18.2)	23	(22.8)	27	(15.6)	0.147^b^
	Parkinson disease	17	(6.2)	10	(9.9)	7	(4.0)	0.069^b^
	Dementia	103	(37.6)	44	(43.6)	59	(34.1)	0.123^b^
Medication causing dysphagia	Antipsychotics	84	(30.7)	36	(35.6)	48	(27.7)	0.177^b^
	Anxiolytics/Hypnotics	69	(25.2)	29	(28.7)	40	(23.1)	0.316^b^
	Anticholinergics	17	(6.2)	7	(6.9)	14	(8.1)	0.817^b^
	Antiemetics/Antiulcer agents	3	(1.1)	3	(3.0)	0	(0)	0.049^b^
	Central muscle relaxants	1	(0.4)	0	(0)	1	(0.6)	1.000^b^

Figures shown as number of patients (%) or median [interquartile range]

a) Mann–Whitney U test (reduced vs normal swallowing function group), b) Fisher’s exact test (reduced vs normal swallowing function group)

BMI: body mass index

### Patient characteristics after propensity score matching

To adjust for potential biases in background factors, propensity score matching was performed, resulting in 72 residents per group. After matching, antipsychotic use was significantly higher in the reduced swallowing function group (28 residents, 38.9%) than in the normal swallowing function group (14 residents, 19.4%) (*P* = 0.017). However, no significant difference in anxiolytic/hypnotic use was observed, with 25 residents (34.7%) in the reduced swallowing function group and 14 (19.4%) in the normal swallowing function group (*P* = 0.060) (Table 2).

**Table 2:** Patient characteristics after propensity score matching

Category		EAT-10 Questionnaire				
		Reduced swallowing function group (n = 72)		Normal swallowing function group (n = 72)		*p*-value
Age		84.5	[76.0–90.0]	87.0	[79.8—91.2]	0.056^a^
Sex	Male	27	(37.5)	21	(29.2)	0.377^b^
	Female	45	(62.5)	51	(70.8)	
BMI (kg/m^2^)		19.5	[17.8—20.9]	19.5	[18.1—21.1]	0.930^a^
Nursing care level	Requiring assistance 1	0	(0)	0	(0)	0.532^b^
	Requiring assistance 2	1	(1.4)	0	(0)	
	Requiring nursing care 1	3	(4.2)	2	(2.8)	
	Requiring nursing care 2	5	(6.9)	9	(12.5)	
	Requiring nursing care 3	20	(27.8)	17	(23.6)	
	Requiring nursing care 4	29	(40.3)	35	(48.6)	
	Requiring nursing care 5	14	(19.4)	9	(12.5)	
Disease causing dysphagia	Stroke	20	(27.8)	14	(19.4)	0.327^b^
	Parkinson disease	6	(8.3)	5	(6.9)	1.000^b^
	Dementia	32	(44.4)	30	(41.7)	0.866^b^
Medication related to dysphagia	Antipsychotics	28	(38.9)	14	(19.4)	0.017^b^
	Anxiolytics/Hypnotics	25	(34.7)	14	(19.4)	0.060^b^
	Anticholinergics	7	(9.7)	5	(6.9)	0.764^b^
	Antiemetics/Antiulcer agents	3	(4.2)	0	(0)	0.245^b^
	Central muscle relaxants	0	(0)	0	(00)	NA

Figures shown as number of patients (%) or median [interquartile range]; NA: Not Applicable

a) Mann–Whitney U test, b) Fisher's exact test

BMI: body mass index

### Main medications used in the reduced swallowing function group

As shown in Table 3, the most commonly used antipsychotics in the reduced swallowing function group were risperidone (11 residents, 39.3%), followed by aripiprazole, quetiapine, and tiapride (each 5 residents, 17.9%). Brotizolam was the most frequently used anxiolytic/hypnotic agent (6 patients, 24.0%).

**Table 3:** Main medications used in the reduced swallowing function group

	Name of medication	No. of users (%)	
Antipsychotics	Risperidone	11	(39.3)
	Aripiprazole	5	(17.9)
	Quetiapine	5	(17.9)
	Tiapride	5	(17.9)
Anxiolytics/Hypnotics	Brotizolam	6	(24.0)
	Nitrazepam	3	(12.0)
	Etizolam	3	(12.0)
	Eszopiclone	3	(12.0)
	Zolpidem	3	(12.0)
Anticholinergics	Trihexyphenidyl	2	(28.6)
	Propiverine	2	(28.6)
	Imidafenacin	1	(14.3)
	Solifenacin	1	(14.3)
Antiemetics/Antiulcer agents	Metoclopramide	3	(100.0)

Figures shown as number of patients (%)

### Medications with a suggested relationship to reduced swallowing function

Antipsychotics and anxiolytics/hypnotics (*p*-value < 0.15 in univariate analysis) were further analyzed using multivariate logistic regression. The results indicated a significant association between antipsychotic use and reduced swallowing function (odds ratio [OR] 2.600, 95% confidence interval [CI], 1.210–5.560; *P* = 0.014) (Table 4).

**Table 4:** Medications with a suggested relationship to reduced swallowing function

Category		Odds ratio	95% confidence interval	*p*-value
Medication related to dysphagia	Antipsychotics	2.600	1.210–5.560	0.014
	Anxiolytics/Hypnotics	2.160	0.996–4.690	0.051

## Discussion

This study examined the prevalence of dysphagia among nursing home residents visited by community pharmacists using the EAT-10 questionnaire, and explored the relationship between medication use and swallowing function in this population. Upon analyzing patient characteristics (in the full population), as shown in Table 1, 101 (36.9%) of 274 residents had an EAT-10 score ≥ 3, classifying them in the reduced swallowing function group. This prevalence was lower than that reported in a previous study (50.2%; 95% CI 33.3–67.2) [15]. This discrepancy may be attributed to differences in assessment methods, as the previous study incorporated videoendoscopic and videofluoroscopic swallowing studies, in addition to the EAT-10 questionnaire, to evaluate swallowing function.

Residents in the reduced swallowing function group were significantly older (median 86.0 years IQR: 79.0–90.0 vs. 83.0 years IQR: 75.0–90.0; *P* = 0.022) and had a lower BMI (median 18.9 kg/m^2^ IQR: 17.6–20.7 vs. 20.3 kg/m^2^ IQR: 18.6–22.5; *P* < 0.001) than those in the normal swallowing function group. These findings align with previous studies indicating that age-related physiological decline in swallowing function and malnutrition increase the risk of dysphagia [16, 17]. This highlights the importance of timely assessment of swallowing function in older and malnourished patients.

After propensity score matching to adjust for background factors, the analysis revealed a significant association between antipsychotic use and reduced swallowing function (OR 2.600, 95% CI, 1.210–5.560; *P* = 0.014). Antipsychotics block dopamine D2 receptors in the striatum of the basal ganglia, leading to extrapyramidal side effects that impair the swallowing muscle function [18]. Additionally, antidopaminergics may reduce substance P levels in the pharynx, thereby suppressing swallowing and cough reflexes and increasing the risk of dysphagia [19]. Given these effects, caution is required when prescribing antipsychotics to patients at a risk of swallowing dysfunction.

In this study, most antipsychotic users in the reduced swallowing function group were administered atypical antipsychotics, including risperidone, aripiprazole, and quetiapine (Table 3). Compared to typical antipsychotics, atypical antipsychotics have a lower reported incidence of extrapyramidal symptoms (20–50%) [20]. However, Sugishita et al. found that 18.8% of older adults using atypical antipsychotics developed dysphagia, with 70% experiencing onset within one day of starting treatment [21]. Similarly, Nozaki et al. reported that dysphagia developed within one week of initiating standard-dose treatment [22]. Based on these findings, patients should be closely monitored for changes in swallowing function, particularly before and after the first week of treatment or dose adjustment of antipsychotics (including atypical antipsychotics).

This study did not find a statistically significant association between anxiolytic/hypnotic use and reduced swallowing function (OR 2.160, 95% CI, 0.996–4.690; *P* = 0.051). However, benzodiazepines were commonly used in the reduced swallowing function group (Table 3), suggesting their potential influence on swallowing function. Uusi-Oukari et al. found that benzodiazepines inhibit neural activity via the GABAA receptors [23]. They have also been shown to bind strongly to the sedative α1 subunit as well as the muscle-relaxing α2, α3, and α5 subunits [24, 25], which may cause relaxation of the swallowing muscles, increasing the risk of aspiration. Given this mechanism, it is essential to monitor changes in swallowing function after administering benzodiazepines and switching to non-benzodiazepine alternatives with lower muscle-relaxing properties, as needed.

To further explore the magnitude of the associations, approximate standardized effect sizes (Cohen’s *d*) were calculated from odds ratios using the method proposed by Chinn [26]. The approximate standardized effect sizes (Cohen’s d) for antipsychotic and anxiolytic/hypnotic use were 0.528 and 0.425, respectively, indicating moderate associations. These findings suggest that, although the association between anxiolytic/hypnotic use and reduced swallowing function did not reach statistical significance (*P* = 0.051), the effect size was sufficiently large to be clinically meaningful. Thus, the possibility of a true association should not be ruled out, and future studies with larger sample sizes are warranted to further clarify the relationship.

This retrospective observational study has several limitations: 1) It was not possible to completely eliminate the influence of confounding factors such as patient history, polypharmacy, and variations in care systems across facilities, as these factors could not be adequately adjusted for in the analysis. Therefore, their potential impacts on the results must be carefully considered. 2) The EAT-10 questionnaire relies on subjective self-assessment, and future research should incorporate objective measures, such as videofluoroscopic swallowing studies, to strengthen the findings. 3) The study population was limited to a specific region and included only facilities that received visits from pharmacists employed by COSMOS CHOUZAI Pharmacy Co., Ltd., which may limit the generalizability of the results to other settings. 4) This study did not establish a direct causal relationship between antipsychotic drug use and reduced swallowing function. Further research is required to investigate the potential causal mechanisms, including the effects of dosage, treatment duration, and interactions with concomitant medications. 5) Due to the retrospective design, information on meal types and standardized dietary assessments, such as the Functional Oral Intake Scale and the Food Intake Level Scale, was unavailable. Future prospective studies should incorporate these assessments to better evaluate the relationship between meals and swallowing function. 6) Furthermore, although the EAT-10 is a widely used subjective screening instrument, its scores may be influenced by the type and consistency of food consumed. However, this study did not collect detailed dietary information, making it difficult to evaluate this potential influence. Future research should consider the impact of food texture on EAT-10 responses. 7) Data regarding whether residents required feeding assistance were not collected. Since feeding assistance may influence swallowing function, future studies should include this variable to enhance the understanding of dysphagia risk factors. 8) Information regarding the use of medications intended to improve swallowing function (e.g., angiotensin-converting enzyme inhibitors, amantadine, cilostazol, and hangekobokuto) was not collected. Future studies should consider these medications as potential confounding factors. 9) No large-scale studies have yet quantified the incidence of drug-induced dysphagia, making it challenging to establish a meaningful a priori sample size calculation. Furthermore, given the retrospective and exploratory nature of this study, an a priori sample size calculation was not performed. Therefore, the possibility of type II error should be considered when interpreting the findings, and future prospective studies with appropriate sample size planning are warranted.

## Conclusions

This study identified a significant association between antipsychotic use and reduced swallowing function among nursing home residents, as assessed by community pharmacists using the EAT-10 questionnaire. Future prospective studies utilizing objective swallowing assessments are needed to clarify the causal relationships between medication use and dysphagia, and to develop suitable interventions.

## Data Availability

No datasets were generated or analysed during the current study.

## Abbreviations

EAT-10: Eating Assessment Tool-10
BMI: Body mass index
IQR,: Interquartile range
OR: Odds ratio
CI: Confidence interval
